# Real-World Effectiveness and Safety of Esomeprazole/Magnesium Hydroxide in Gastroesophageal Reflux Disease: Results from a Multicenter Prospective Observational Study

**DOI:** 10.3390/jpm16100497

**Published:** 2026-09-25

**Authors:** Seung Han Kim, Na Young Kim

**Affiliations:** 1 Department of Internal Medicine, Korea University Guro Hospital, Korea University College of Medicine, Seoul 08308, Republic of Korea; 2 Hanmi Pharmaceutical Co., Ltd., Seoul 05545, Republic of Korea; skyko7@hanmi.co.kr

**Keywords:** gastroesophageal reflux disease, esomeprazole, magnesium hydroxide, proton pump inhibitor, real-world evidence, patient-reported outcomes, observational study

## Abstract

**Background/Objectives**: Proton pump inhibitors (PPIs) are the mainstay treatment for gastroesophageal reflux disease (GERD); however, variability in treatment response and suboptimal symptom control pose clinical challenges. This study aimed to evaluate the real-world effectiveness and safety of esomeprazole/magnesium hydroxide in patients with GERD using a structured symptom assessment. **Methods**: This multicenter, prospective, non-interventional study included patients with GERD who were prescribed esomeprazole/magnesium hydroxide in routine clinical practice. Symptoms were assessed at baseline and at weeks 4 and 8. The prespecified primary endpoint was the protocol-defined responder rate at week 4; a post hoc sensitivity analysis required a decrease in total symptom score. **Results**: A total of 5228 patients were included in the effectiveness analysis. Protocol-defined responder rates were 98.4% and 96.2% at weeks 4 and 8, respectively. In the stricter sensitivity analysis, responder rates were 91.1% and 93.4%, respectively. The median total symptom score decreased from 6.0 at baseline to 3.0 at week 4 and 1.0 at week 8 (nominal *p* < 0.0001). Symptom improvement was observed across erosive esophagitis, non-erosive GERD, and maintenance-therapy subgroups. Adverse events occurred in 0.7% of patients. **Conclusions**: Esomeprazole/magnesium hydroxide use was associated with symptom improvement over 8 weeks in routine practice. Because this uncontrolled observational study cannot establish causality, the findings should be interpreted as descriptive real-world evidence.

## 1. Introduction

Gastroesophageal reflux disease (GERD) is a highly prevalent condition that considerably impairs the quality of life and imposes a substantial healthcare burden worldwide [1,2,3,4]. Proton pump inhibitors (PPIs) are the cornerstone of GERD treatment [5,6,7]; however, variability in treatment response and suboptimal symptom control in some patients remains an ongoing challenge in clinical practice [8,9,10].

Esomeprazole, a widely used PPI, suppresses gastric acid secretion through irreversible inhibition of the H^+^/K^+^-ATPase enzyme [11,12]. However, conventional PPI formulations require activation in an acidic environment and may exhibit variability in the onset and consistency of acid suppression [11]. Therefore, a formulation combining esomeprazole with magnesium hydroxide has been developed [13]. Magnesium hydroxide acts as a buffering agent that increases intragastric pH and protects esomeprazole from acid degradation, potentially facilitating more efficient drug absorption and consistent pharmacodynamic effects [14].

Despite these pharmacological advantages, evidence on the performance of esomeprazole/magnesium hydroxide across the heterogeneous patients treated in routine practice remains limited. Real-world evaluation is relevant because patients may receive the formulation for erosive esophagitis, symptomatic non-erosive GERD, or maintenance therapy, and because prior treatment exposure and physician-selected dosing may influence observed outcomes. In addition, many observational studies have used heterogeneous and nonstandardized symptom assessments, limiting comparison across studies [15].

Therefore, this study aimed to evaluate the real-world effectiveness and safety of esomeprazole/magnesium hydroxide in patients with GERD using a structured symptom assessment and to explore whether outcomes were consistent across clinically relevant patient subgroups.

## 2. Methods

### 2.1. Study Design and Setting

This multicenter, prospective, noninterventional, observational study was conducted in routine clinical practice across 166 centers in Korea between August 2022 and July 2024. Patients were consecutively enrolled if they were prescribed esomeprazole/magnesium hydroxide, based on the clinical judgment of the treating physician. No intervention or treatment allocation was mandated by the study protocol. Ethics approval details can be found in Table S1.

### 2.2. Study Population

Eligible patients were adults (>=19 years) diagnosed with GERD and prescribed esomeprazole/magnesium hydroxide for treatment of erosive esophagitis, symptomatic treatment of non-erosive GERD, or maintenance therapy to prevent recurrence. Consecutive enrollment was used to reduce discretionary selection; however, because treatment had already been selected by the physician, the study population may not represent untreated patients or patients receiving alternative therapies.

The key exclusion criteria were hypersensitivity to the study drug components, pregnancy or lactation, concomitant use of contraindicated medications (e.g., atazanavir, nelfinavir, and rilpivirine), severe renal impairment, and active diarrhea.

The effectiveness analysis set included patients who underwent at least one postbaseline symptom assessment, whereas the safety analysis set included all patients who received at least one dose of the study medication.

### 2.3. Treatment and Follow-Up

Patients received esomeprazole/magnesium hydroxide at doses of 20/350 mg or 40/350 mg once daily, according to approved indications and physician discretion. Treatment strategies followed routine clinical practice, including step-down or maintenance therapy, where appropriate.

Patients were followed for approximately 8 weeks, with assessments at baseline and at weeks 4 and 8. When an in-person visit was not feasible, the follow-up assessment was conducted by telephone. At each contact, the investigators recorded symptom assessments and safety information reported by the patient or identified during routine care.

### 2.4. Data Collection and Variables

Baseline data included demographic characteristics, prior GERD-related medications (within 3 months), comorbidities (including severe hepatic impairment and allergic conditions), and concomitant medications.

GERD symptoms were assessed using a structured symptom scoring system to evaluate heartburn, acid regurgitation, dysphagia, and epigastric pain. Each symptom was scored on a 4-point scale (0–3), with higher scores indicating greater severity.

### 2.5. Outcome Measures

The primary endpoint was the protocol-defined GERD symptom responder rate at week 4. A protocol-defined responder was a patient whose total symptom score decreased or remained unchanged from baseline, representing improvement or non-worsening. Because inclusion of unchanged scores could inflate the apparent response, a post hoc sensitivity analysis used a stricter definition requiring a decrease in total symptom score.

Secondary endpoints included the protocol-defined responder rate at week 8, changes in total and individual symptom-domain scores from baseline at weeks 4 and 8, and descriptive subgroup analyses by clinical indication, prior GERD medication use, and initial dose.

Safety endpoints included the incidence of adverse events (AEs), adverse drug reactions (ADRs), serious adverse events (SAEs), and serious adverse drug reactions (SADRs). An AE was any untoward medical occurrence after treatment initiation, irrespective of causal relationship. An ADR was an AE for which a causal relationship to esomeprazole/magnesium hydroxide could not be excluded based on the investigator’s clinical assessment. Seriousness and severity were evaluated separately; severity was categorized as mild, moderate, or severe, whereas seriousness was determined using standard regulatory criteria such as death, life-threatening events, hospitalization, disability, or other medically important conditions.

### 2.6. Statistical Analyses

Continuous variables are summarized as means ± standard deviations or as medians with interquartile ranges (IQRs), whereas categorical variables are presented as numbers and percentages.

Changes in symptom scores from baseline were evaluated separately at weeks 4 and 8 using paired *t*-tests or Wilcoxon signed-rank tests, depending on the distribution assessed with the Shapiro–Wilk test. These prespecified visit-specific comparisons were not intended as a formal longitudinal model. Because multiple secondary and subgroup comparisons were exploratory, no multiplicity adjustment was applied and their *p*-values should be interpreted as nominal.

GERD responder rates were calculated with 95% confidence intervals (CIs). In the full analysis set, patients with a missing symptom assessment at a given postbaseline visit were classified as nonresponders. A complete-case sensitivity analysis was also performed using only patients with an available assessment at the corresponding visit. CIs were estimated with the Wald method when the normal approximation was appropriate and the exact Clopper–Pearson method otherwise.

All statistical analyses were performed using the SAS software (version 9.4). Statistical significance was defined as a two-sided *p*-value < 0.05.

Initial-dose groups were compared descriptively because treatment dose was selected by the treating physician rather than randomized. Between-group baseline comparisons used a two-sample *t*-test or Wilcoxon rank-sum test for continuous variables and a chi-squared test or Fisher’s exact test for categorical variables, as appropriate. These comparisons were exploratory and do not support causal conclusions about relative dose effectiveness.

### 2.7. Ethical Considerations

This study was conducted in accordance with the principles of the Declaration of Helsinki. The study protocol was approved by the Public Institutional Review Board designated by the Ministry of Health and Welfare, Republic of Korea (approval code: P01-202208-01-035; date of approval: 25 August 2022) or the institutional review board at each participating center. Written informed consent was obtained from all patients before enrollment.

## 3. Results

### 3.1. Patient Disposition and Baseline Characteristics

A total of 5307 patients were enrolled in this study. After applying the predefined inclusion criteria, 5283 patients were included in the safety analysis set and 5228 in the effectiveness analysis set (Figure 1). The most common reason for exclusion from the effectiveness analysis was the absence of post-baseline symptom assessment.

The baseline demographic and clinical characteristics are presented in Table 1. In the safety analysis set, mean age was 62.4 years in the 20/350 mg group and 60.3 years in the 40/350 mg group (nominal *p* < 0.0001). Sex, height, weight, body mass index, smoking, and alcohol consumption did not differ materially between initial-dose groups (all nominal *p* > 0.05). Because dose selection was based on clinical judgment and the clinical-indication distribution was highly unbalanced between dose groups, these comparisons do not exclude residual confounding.

### 3.2. Effectiveness Outcomes

Using the prespecified definition of improvement or no worsening, the GERD responder rate was 98.4% (95% CI, 98.0–98.7) at week 4 and 96.2% (95% CI, 95.7–96.7) at week 8 (Table 2 and Figure 2). Under the stricter sensitivity definition requiring a decrease in total symptom score, responder rates were 91.1% (95% CI, 90.4–91.9) and 93.4% (95% CI, 92.8–94.1), respectively. The corresponding strict-definition rates were 90.5% and 95.3% in the 20/350 mg group and 91.8% and 91.5% in the 40/350 mg group at weeks 4 and 8. Because dose selection was nonrandom and baseline age differed between groups, these dose-specific estimates are descriptive.

At week 4, symptom assessments were available for 5188 of 5228 patients (99.2%); at week 8, they were available for 5048 patients (96.6%). Under the protocol-defined responder criterion, available-case responder rates were 99.1% (5142/5188) at week 4 and 99.6% (5029/5048) at week 8. The full-set analysis classified the 40 and 180 patients with missing assessments at weeks 4 and 8, respectively, as nonresponders.

The changes in GERD symptom scores over time are summarized in Table 3. The median total symptom score decreased from 6.0 (IQR, 4.0–7.0) at baseline to 3.0 (IQR, 1.0–4.0) at week 4, corresponding to a median reduction of −3.0. Further improvement was observed at week 8, with the median score decreasing to 1.0 (IQR, 0.0–2.0) and a median change of −4.0 from baseline. These reductions were statistically significant at all time points (*p* < 0.0001).

Improvements were observed in all symptom domains. Heartburn and acid-regurgitation scores decreased from medians of 2.0 at baseline to 1.0 at week 4 and 0.0 at week 8. Median dysphagia and epigastric-pain scores also decreased to 0.0 by week 4 and remained at 0.0 at week 8. All visit-specific comparisons yielded nominal *p* < 0.0001; these exploratory *p*-values were not adjusted for multiple comparisons.

In analyses by clinical indication, the protocol-defined responder rates at weeks 4 and 8 were 98.4% and 94.9% among patients treated for erosive esophagitis (n = 2389), 98.2% and 97.1% among patients with non-erosive GERD (n = 1971), and 98.6% and 97.7% among those receiving maintenance therapy (n = 868). Median total symptom scores decreased from 6.0 at baseline to 3.0 at week 4 and 1.0 at week 8 in both the erosive-esophagitis and maintenance-therapy groups; in the non-erosive GERD group, the corresponding medians were 5.0, 2.0, and 1.0. These descriptive findings indicate improvement across the three clinical indications without establishing comparative effectiveness between them.

Subgroup analyses showed that patients with prior use of GERD medications had a lower likelihood of achieving a response than those who had not received prior treatment (Figure 3). Multivariable analysis demonstrated that prior medication use was significantly associated with treatment response at week 4 (odds ratio, 0.60; 95% CI, 0.39–0.94; *p* = 0.0244). Nevertheless, high responder rates were observed in both groups.

### 3.3. Safety Outcomes

Safety outcomes are summarized in Table 4. Among 5283 patients in the safety analysis, 39 (0.7%) experienced 50 AEs. Severity categories describe events rather than mutually exclusive patients: 44 events were mild, two were moderate, and four were severe; therefore, patient counts across severity categories need not sum to the number of patients with any AE because a patient could experience events of different severities.

ADRs were reported in 15 patients (0.3%), comprising 19 events (18 mild and one moderate). SAEs occurred in five patients (0.1%), and no SADRs were identified. One death occurred during follow-up but was assessed by the investigator as unrelated to the study medication. Twenty patients discontinued treatment because of AEs, including 14 who discontinued because of ADRs.

The most frequently reported AEs are listed in Table 5. Disorders of the gastrointestinal and nervous systems were the most common, with each occurring in a small proportion of patients. The most frequently reported individual events were diarrhea and headache. A similar distribution was observed for the ADRs. Overall, no unexpected safety concerns were reported.

## 4. Discussion

In this large, multicenter, prospective observational study, esomeprazole/magnesium hydroxide use was associated with improvement in GERD symptoms during approximately 8 weeks of routine care. The protocol-defined responder rates were 98.4% at week 4 and 96.2% at week 8, while a stricter sensitivity analysis requiring an actual decrease in total symptom score yielded rates of 91.1% and 93.4%, respectively. The latter analysis confirms that the main pattern was not explained solely by classifying unchanged scores as responses.

A key strength of this study was the inclusion of patients treated for erosive esophagitis, non-erosive GERD, and maintenance therapy. Symptom scores decreased in all three groups, and protocol-defined responder rates remained high. However, these subgroup analyses were descriptive, group sizes and dose distributions were markedly unbalanced, and this study was not designed for formal comparative inference between clinical indications.

Subgroup analyses based on prior GERD medication use revealed that patients with prior treatment had a lower likelihood of responding than those without prior treatment. Nevertheless, high responder rates were observed in both groups, indicating that clinically meaningful symptom improvement was achieved irrespective of prior treatment status. These findings are particularly relevant because treatment-experienced patients represent a substantial proportion of those encountered in routine clinical practice.

The observed symptom improvement is pharmacologically plausible because magnesium hydroxide can buffer gastric acid and protect esomeprazole from acid degradation, while esomeprazole provides sustained acid suppression [13]. Nevertheless, plausibility does not establish treatment causality. Without a concurrent control group, regression to the mean, natural symptom fluctuation, concomitant care, adherence effects, and patient or investigator expectations cannot be separated from the treatment-associated change.

The observed AE incidence was low and most reported events were mild. This finding should be interpreted in the context of the approximately 8-week observational follow-up and routine-practice safety ascertainment, which may not capture uncommon, delayed, or unreported events. The absence of SADRs in this cohort does not exclude rare risks or risks associated with longer treatment exposure.

This study has several limitations. First, treatment was not randomized and there was no comparator group; consequently, the observed within-patient changes cannot be attributed solely to esomeprazole/magnesium hydroxide. Second, physician-directed prescribing may have introduced selection bias and confounding by indication, particularly in comparisons between initial doses. Third, symptoms were investigator-administered rather than confirmed by objective reflux monitoring. Fourth, 40 patients lacked a week-4 assessment and 180 lacked a week-8 assessment; although the primary full-set analysis treated missing visits as nonresponse and an available-case analysis gave similar conclusions, bias remains possible if missingness was related to outcome. Fifth, multiple secondary and subgroup comparisons were exploratory and were not adjusted for multiplicity. Sixth, the short follow-up and routine-practice reporting may underestimate uncommon or delayed AEs. Finally, the manufacturer sponsored this study and participated in its design and statistical planning. Although the authors retained responsibility for data interpretation and the decision to publish, sponsor involvement remains a potential source of bias.

Within these constraints, the large prospective cohort provides descriptive real-world evidence on symptom trajectories and short-term safety after initiation of esomeprazole/magnesium hydroxide across several clinical indications.

In conclusion, esomeprazole/magnesium hydroxide use was associated with improved GERD symptom scores over approximately 8 weeks in routine clinical practice, including under a stricter responder definition and across erosive esophagitis, non-erosive GERD, and maintenance-therapy subgroups. The uncontrolled observational design precludes causal or comparative conclusions, and the short-term safety findings should not be extrapolated to uncommon or long-latency events.

## Figures and Tables

**Figure 1:**
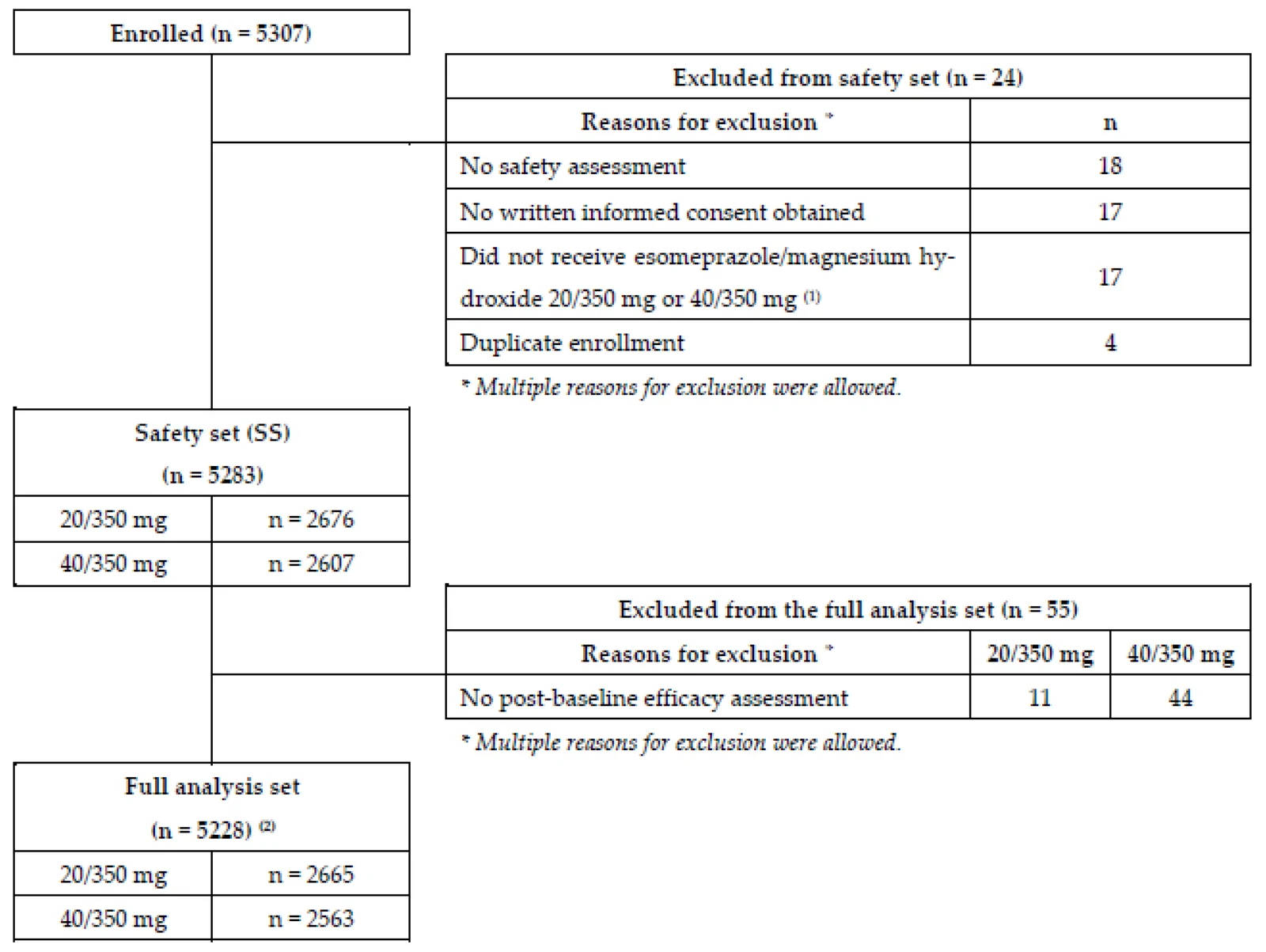
Study flow diagram. ^(1)^ Did not receive esomeprazole/magnesium hydroxide 20/350 mg or 40/350 mg: defined as patients with missing dosing information or a treatment initiation date after the end of the follow-up period. ^(2)^ Patients were classified based on the initial dose group if dose changes occurred during the study period.

**Figure 2:**
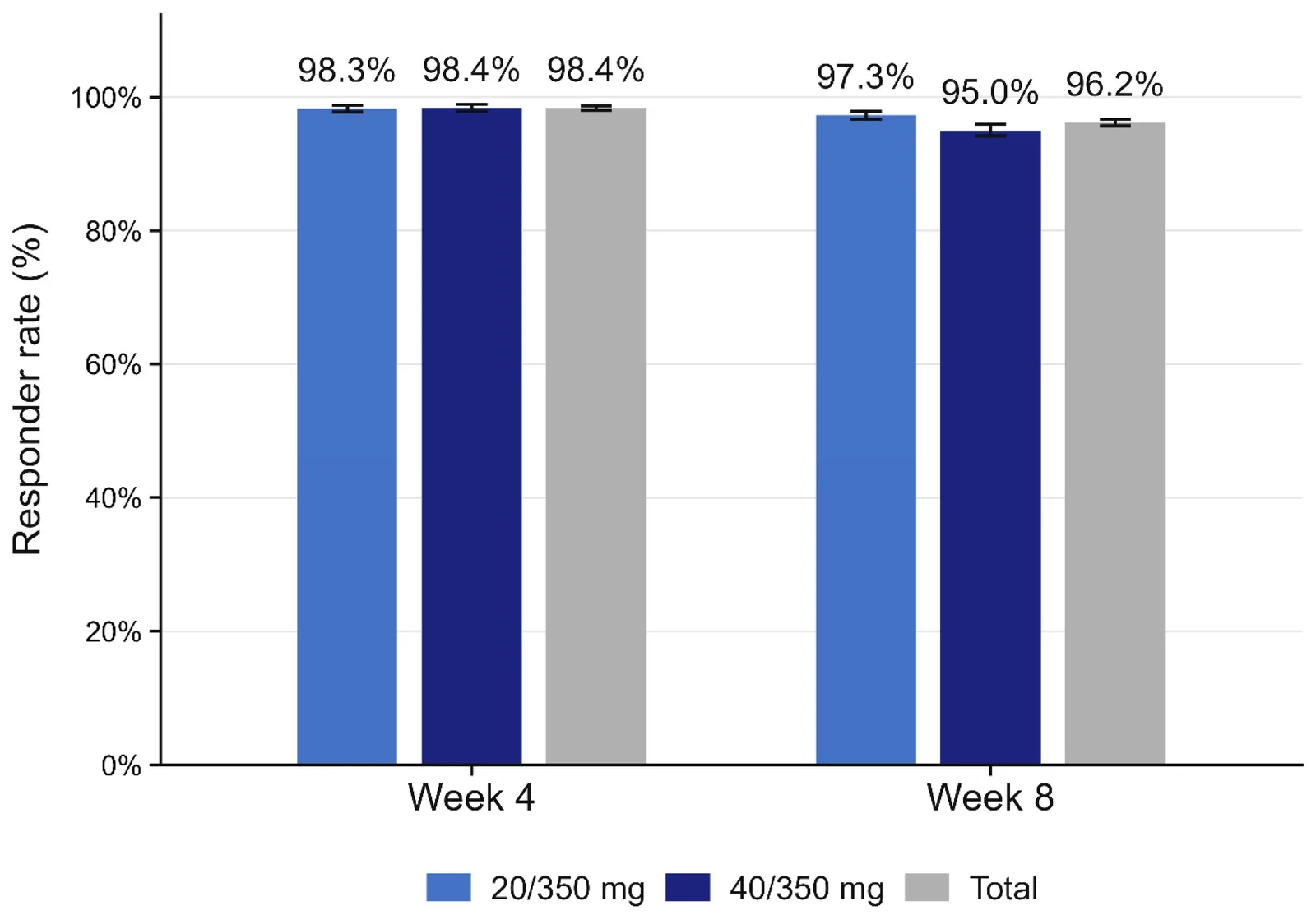
Responder rates at weeks 4 and 8. The proportion of patients achieving symptom response remained consistently high over time, demonstrating sustained effectiveness of esomeprazole/magnesium hydroxide in routine clinical practice.

**Figure 3:**
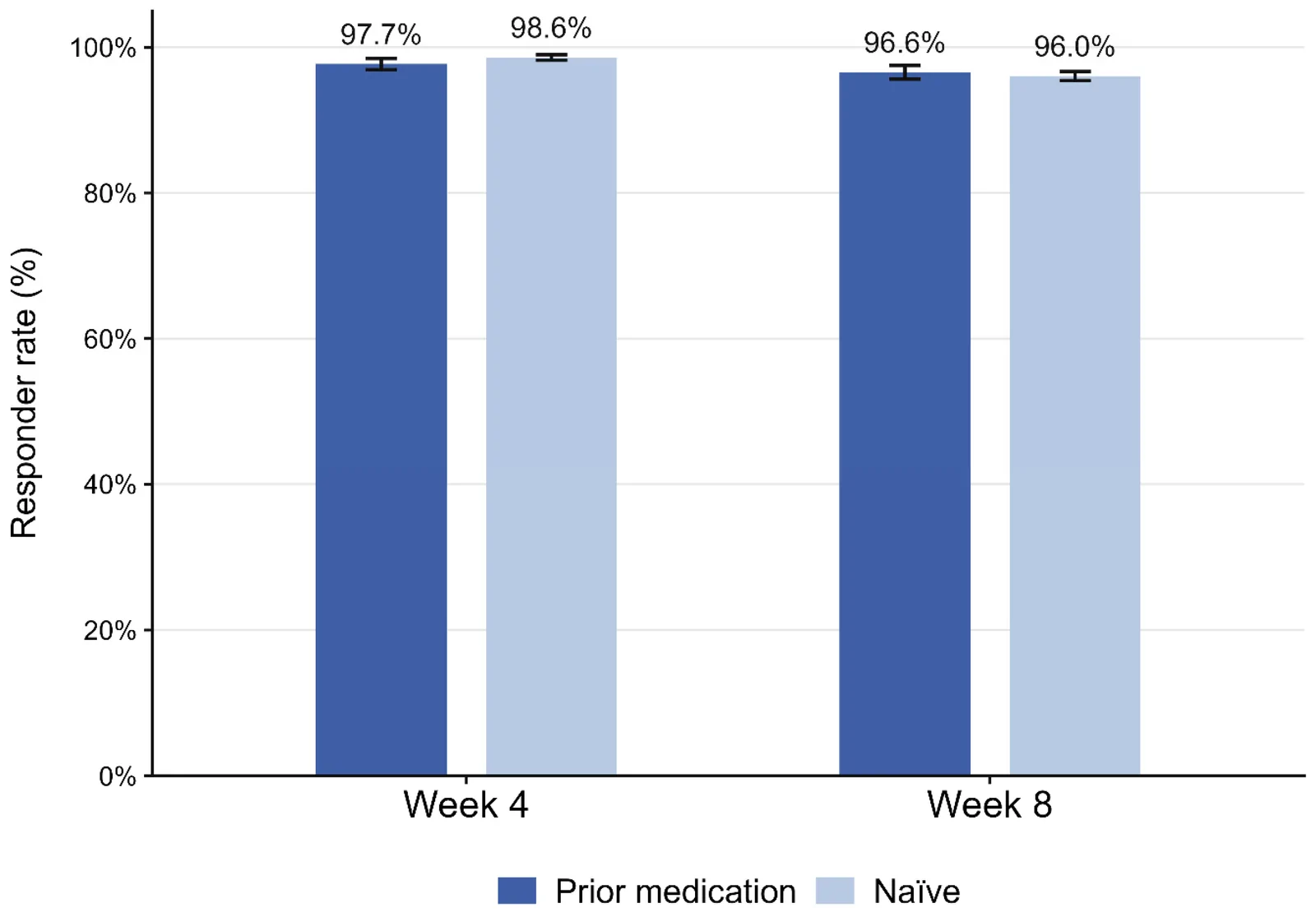
Responder rates according to prior gastroesophageal reflux disease (GERD) medication use. Responder rates at weeks 4 and 8 are shown for patients with and without prior GERD medication use. Although prior medication use was associated with a lower likelihood of response at week 4, high response rates were consistently observed in both groups, indicating the maintained effectiveness of esomeprazole/magnesium hydroxide in treatment-experienced patients.

**Table 1:** Demographic characteristics.

	Esomeprazole Magnesium Trihydrate/Magnesium Hydroxide					
	20/350 mg (n = 2676)		40/350 mg (n = 2607)		Total (n = 5283)	
Age (years)	62.4	(14.4)	60.3	(13.7)	61.4	(14.1)
Male (%)	1295	(48.4)	1316	(50.5)	2611	(49.4)
Height (cm)	162.4	(9.3)	162.9	(9.1)	162.7	(9.2)
Weight (kg)	64.0	(11.9)	64.6	(12.0)	64.3	(12.0)
Body mass index (kg/m^2^)	24.2	(3.4)	24.2	(3.3)	24.2	(3.4)
≥25 and ≤30, n (%)	315	(11.8)	366	(14.0)	681	(12.9)
>30, n (%)	58	(2.2)	62	(2.4)	120	(2.3)
Smoking, n (%)	389	(14.5)	379	(14.5)	768	(14.5)
Alcohol consumption, n (%)	691	(25.8)	680	(26.1)	1371	(26.0)

n: number of patients; %: percentage of patient. Values are presented as means (standard deviations) except for sex, body mass index group, smoking, and alcohol consumption, which are presented as n (%).

**Table 2:** Summary of GERD responder rates (full analysis set).

	Esomeprazole Magnesium Trihydrate/Magnesium Hydroxide					
	20/350 mg (n = 2665)		40/350 mg (n = 2563)		Total (n = 5228)	
	n (%)	95% CI	n (%)	95% CI	n (%)	95% CI
Gastroesophageal reflux disease responder						
-Week 4	2620 (98.3)	[97.8, 98.8]	2522 (98.4)	[97.9, 98.9]	5142 (98.4)	[98.0, 98.7]
-Week 8	2593 (97.3)	[96.7, 97.9]	2436 (95.0)	[94.2, 95.9]	5029 (96.2)	[95.7, 96.7]

n: number of patients; %: responder rate; CI, confidence interval. 95% CIs were estimated using the Wald method when normal approximation was adequate; otherwise, the exact Clopper–Pearson method was applied.

**Table 3:** Summary of GERD symptom scores (full analysis set).

	Esomeprazole Magnesium Trihydrate/Magnesium Hydroxide								
	20/350 mg (n = 2665)			40/350 mg (n = 2563)			Total (n = 5228)		
	Median	(Q1, Q3)	*p*-Value ^a^	Median	(Q1, Q3)	*p*-Value ^a^	Median	(Q1, Q3)	*p*-Value ^a^
Total symptoms score									
-Baseline	5.0	(3.0, 7.0)		6.0	(4.0, 8.0)		6.0	(4.0, 7.0)	
-Week 4	2.0	(1.0, 4.0)		3.0	(2.0, 4.0)		3.0	(1.0, 4.0)	
Difference from baseline	−2.0	(−4.0, −1.0)	<0.0001 ^#^	−3.0	(−4.0, −2.0)	<0.0001 ^#^	−3.0	(−4.0, −1.0)	<0.0001 ^#^
-Week 8	1.0	(0.0, 2.0)		1.0	(0.0, 2.0)		1.0	(0.0, 2.0)	
Difference from baseline	−4.0	(−6.0, −2.0)	<0.0001 ^#^	−5.0	(−6.0, −3.0)	<0.0001 ^#^	−4.0	(−6.0, −2.0)	<0.0001 ^#^
Score of heartburn									
-Baseline	2.0	(1.0, 2.0)		2.0	(1.0, 2.0)		2.0	(1.0, 2.0)	
-Week 4	1.0	(0.0, 1.0)		1.0	(1.0, 1.0)		1.0	(0.0, 1.0)	
Difference from baseline	−1.0	(−1.0, 0.0)	<0.0001 ^#^	−1.0	(−1.0, 0.0)	<0.0001 ^#^	−1.0	(−1.0, 0.0)	<0.0001 ^#^
-Week 8	0.0	(0.0, 1.0)		0.0	(0.0, 1.0)		0.0	(0.0, 1.0)	
Difference from baseline	−1.0	(−2.0, −1.0)	<0.0001 ^#^	−1.0	(−2.0, −1.0)	<0.0001 ^#^	−1.0	(−2.0, −1.0)	<0.0001 ^#^
Score of acid regurgitation									
-Baseline	2.0	(1.0, 2.0)		2.0	(1.0, 2.0)		2.0	(1.0, 2.0)	
-Week 4	1.0	(0.0, 1.0)		1.0	(0.0, 1.0)		1.0	(0.0, 1.0)	
Difference from baseline	−1.0	(−1.0, 0.0)	<0.0001 ^#^	−1.0	(−1.0, 0.0)	<0.0001 ^#^	−1.0	(−1.0, 0.0)	<0.0001 ^#^
-Week 8	0.0	(0.0, 1.0)		0.0	(0.0, 1.0)		0.0	(0.0, 1.0)	
Difference from baseline	−1.0	(−2.0, −1.0)	<0.0001 ^#^	−1.0	(−2.0, −1.0)	<0.0001 ^#^	−1.0	(−2.0, −1.0)	<0.0001 ^#^
Score of dysphagia									
-Baseline	1.0	(0.0, 2.0)		1.0	(0.0, 2.0)		1.0	(0.0, 2.0)	
-Week 4	0.0	(0.0, 1.0)		0.0	(0.0, 1.0)		0.0	(0.0, 1.0)	
Difference from baseline	0.0	(−1.0, 0.0)	<0.0001 ^#^	0.0	(−1.0, 0.0)	<0.0001 ^#^	0.0	(−1.0, 0.0)	<0.0001 ^#^
-Week 8	0.0	(0.0, 0.0)		0.0	(0.0, 0.0)		0.0	(0.0, 0.0)	
Difference from baseline	0.0	(−1.0, 0.0)	<0.0001 ^#^	−1.0	(−2.0, 0.0)	<0.0001 ^#^	−1.0	(−1.0, 0.0)	<0.0001 ^#^
Score of epigastric pain									
-Baseline	1.0	(0.0, 2.0)		2.0	(1.0, 2.0)		1.0	(1.0, 2.0)	
-Week 4	0.0	(0.0, 1.0)		1.0	(0.0, 1.0)		0.0	(0.0, 1.0)	
Difference from baseline	−1.0	(−1.0, 0.0)	<0.0001 ^#^	−1.0	(−1.0, 0.0)	<0.0001 ^#^	−1.0	(−1.0, 0.0)	<0.0001 ^#^
-Week 8	0.0	(0.0, 0.0)		0.0	(0.0, 0.0)		0.0	(0.0, 0.0)	
Difference from baseline	−1.0	(−1.0, 0.0)	<0.0001 ^#^	−1.0	(−2.0, −1.0)	<0.0001 ^#^	−1.0	(−2.0, 0.0)	<0.0001 ^#^

^a^ Based on the Shapiro–Wilk normality test, differences from baseline were tested using a paired *t*-test or Wilcoxon signed-rank test (^#^). *p*-values are nominal and were not adjusted for multiple comparisons. IQR, interquartile range.

**Table 4:** Summary of safety outcomes (safety analysis set).

	Esomeprazole Magnesium Trihydrate/Magnesium Hydroxide								
	20/350 mg (n = 2676)			40/350 mg (n = 2607)			Total (n = 5283)		
	n	(%)	No. of events	n	(%)	No. of events	n	(%)	No. of events
AE	15	(0.6)	22	24	(0.9)	28	39	(0.7)	50
Mild	11	(0.4)	16	24	(0.9)	28	35	(0.7)	44
Moderate	2	(0.1)	2	0	(0.0)	0	2	(0.0)	2
Severe	4	(0.1)	4	0	(0.0)	0	4	(0.1)	4
Leading to discontinuation	8	(0.3)	10	12	(0.5)	14	20	(0.4)	24
ADR	4	(0.1)	6	11	(0.4)	13	15	(0.3)	19
Mild	4	(0.1)	5	11	(0.4)	13]	15	(0.3)	18
Moderate	1	(0.0)	1	0	(0.0)	0	1	(0.0)	1
Severe	0	(0.0)	0	0	(0.0)	0	0	(0.0)	0
Leading to discontinuation	4	(0.1)	6	10	(0.4)	12	14	(0.3)	18
SAE	5	(0.2)	5	0	(0.0)	0	5	(0.1)	5
Leading to discontinuation	3	(0.1)	3	0	(0.0)	0]	3	(0.1)	3
Deaths	1	(0.0)	1	0	(0.0)	0]	1	(0.0)	1
SADR	0	(0.0)	0	0	(0.0)	0	0	(0.0)	0

n, number of patients; %, percentage of patients; No. of events, number of events. Mild, moderate, and severe are event-level severity categories; a patient may contribute events to more than one category. AE, adverse event; ADR, adverse drug reaction; SAE, serious adverse event; SADR, serious adverse drug reaction.

**Table 5:** Summary of the most frequently reported AEs, ADRs (safety analysis set).

	Esomeprazole Magnesium Trihydrate/Magnesium Hydroxide					
	20/350 mg (n = 2676)		40/350 mg (n = 2607)		Total (n = 5283)	
	n (%)	Frequency of AEs	n (%)	Frequency of AEs	n (%)	Frequency of AEs
Most frequently reported AEs by system organ class and preferred term ^a^						
Gastrointestinal disorders	6 (0.2)	8	9 (0.4)	9	15 (0.3)	17
Diarrhea	2 (0.1)	2	4 (0.2)	4	6 (0.1)	6
Nervous system disorders	6 (0.2)	6	5 (0.2)	5	11 (0.2)	11
Headache	1 (0.0)	1	3 (0.1)	3	4 (0.1)	4
Most frequently reported ADRs by system organ class and preferred term ^b^						
Gastrointestinal disorders	3 (0.1)	4	5 (0.2)	5	8 (0.2)	9
Diarrhea	2 (0.1)	2	4 (0.2)	4	6 (0.1)	6
Nervous system disorders	2 (0.1)	2	5 (0.2)	5	7 (0.1)	7
Headache	1 (0.0)	1	3 (0.1)	3	4 (0.1)	4

n: number of patients; %: percentage of patients; AE, adverse event; ADR, adverse drug reaction. ^a^ ≥ 0.1%. ^b^ ≥ 0.1%.

## Data Availability

Data may be available from the corresponding author upon reasonable request, subject to approval by the study sponsor.

## Supplementary Materials

The following supporting information can be downloaded at: https://www.mdpi.com/article/10.3390/jpm16100497/s1, Table S1: Ethics Approval Details for the HM-ESOP-OS-2201 Study.

## Abbreviations

The following abbreviations are used in this manuscript:

PPI	Proton pump inhibitor
GERD	Gastroesophageal reflux disease
AE	Adverse event
ADR	Adverse drug reaction
SAE	Serious adverse event
SADR	Serious adverse drug reaction
CI	Confidence interval
IQR	Interquartile range
