# Supplementary material for: Real-World Effectiveness and Safety of Esomeprazole/Magnesium Hydroxide in Gastroesophageal Reflux Disease: Results from a Multicenter Prospective Observational Study
Source: J Pers Med. 2026 Sep 25;16(10):497. doi: 10.3390/jpm16100497 (PMC13705982; doi:10.3390/jpm16100497)
Supplement: Supplementary file 1 [file jpm-16-00497-s001.zip › jpm-4488827-supplementary.pdf]

Table S1. Ethics Approval Details for the HM-ESOP-OS-2201 Study

No	Affiliation	Name	Approval code	Approval date (YYYY-MM-DD)
1	Kim Kyung-ho Internal Medicine Clinic	Kyung-ho Kim	P01-202208-01-035 (Public Institutional Review Board)	2022-08-25
2	Dadae Ansim Internal Medicine Clinic	Eun-jeong Kim		
3	Yonsei Sok-pyeon-han Internal Medicine Clinic	Min-seon Park		
4	Lim Tae-jin Internal Medicine Clinic	Tae-jin Lim		
5	365 Kyungdae Union Internal Medicine Clinic	Sun-young Moon		
6	Lee Jong-ik Internal Medicine Clinic	Jong-ik Lee		
7	Park Sang-bae Internal Medicine Clinic	Sang-bae Park		
8	Hansarang Clinic	Mi-ok Oh		
9	Shinhan Yonsei Internal Medicine Clinic	Jeong-ho Cho		
10	Asan Bareun Internal Medicine Clinic	Ju-hyeon Jeon		
11	Hyocheon Union Internal Medicine Clinic	Seong-cheol Park		
12	Dongyang Good Morning Radiology Clinic	Jin-hee Lee		
13	Cho Gil-ho Clinic	Gil-ho Cho		
14	Simin Clinic	In-su Jeong		
15	Sintongbangtong Clinic	Min-seok Kim		
16	Goryeo Dream Internal Medicine Clinic	Dong-hoon Lee		
17	Goryeo Dream Internal Medicine Clinic	Jeong-hwan Lee		
18	Kim Du-kyeong Internal Medicine Clinic	Du-kyeong Kim		
19	Pangyo Yonsei Internal Medicine Clinic	Yeong-gyun Kim		
20	Park Yong-hyeon Internal Medicine Clinic	Yong-hyeon Park	P01-202208-01-035	2022-08-25

21	Seonwoo ENT Clinic	Jong-seon Yoo	(Public Institutional Review Board)	
22	Yugu Seongshim Clinic	Tae-hwan Ko		
23	Healthy Sok Internal Medicine Clinic	Jun-cheol Yoon		
24	Hoon Internal Medicine Clinic	Dong-hoon Shin		
25	Gajok Sarang Clinic	Woo-jong Kim		
26	Wellbeing Soo Clinic	Jeong-deok Hwang		
27	Park Jae-seok Internal Medicine Clinic	Jae-seok Park		
28	Saebit Tunteun Clinic	Gil-sun Park		
29	Nanum Internal Medicine Clinic	Jun-gwang Son		
30	Samsung Family Medicine Clinic	Haeng-hoon Lee		
31	Jeil Clinic	Byeong-jeong Kim		
32	Kind Neurosurgery Clinic	Jin-young Kim		
33	Choi Kang-sik Internal Medicine Clinic	Kang-sik Choi		
34	Okpo Shinsegae Union Clinic	Jae-pil Lee		
35	Geumwang Samsung Union Clinic	Gyu-hwan Yang		
36	Yoo Kyung-wan Internal Medicine Clinic	Kyung-wan Yoo		
37	Daegu Samsun Hospital	In-guk Lee		
38	Comfortable Internal Medicine Clinic	Seong-min Han		
39	Seongmo Best Internal Medicine Clinic	Sun-hoo Kim		
40	Smile Internal Medicine Clinic	Sang-woo Park		
41	Choi Internal Medicine Clinic	Song-i Kim		
42	Jang Gyu-man Internal Medicine Clinic	Gyu-man Jang		
43	Jeong Jin Internal Medicine Clinic	Jin-mo Jeong	P01-202208-01-035	2022-08-25

44	Howon Jeil Clinic	Chang-eop Park	(Public Institutional Review Board)	
45	365 Janggok Pyeonhan Internal Medicine Clinic	Young-jip Kim		
46	Prime Seongmo Internal Medicine Clinic	Soon-seop Kim		
47	Park Jae-seok Internal Medicine Clinic	Jae-seok Park		
48	Wondang Yonsei Medical Clinic	Seong-hwan Kang		
49	Bagae Clinic	Jeong-gyu Yang		
50	Myeongji Internal Medicine Clinic	Seung-min Jeon		
51	Cheongpyeong Yonsei Clinic	Jong-hwa Kim		
52	Hanbit Family Clinic	Jae-gyun Jeong		
53	Yangpyeong Medical Foundation	Hong-deok Kim		
54	Lee Jae-chun Internal Medicine Clinic	Jae-chun Lee		
55	Dongtan Seoul Asan Internal Medicine Clinic	Jeong-hyeon Park		
56	Da Vinci Hospital	San-gyeong Kang		
57	Kim Kyung-a Family Medicine Clinic	Kyung-a Kim		
58	Hanjoo Clinic	Young Kim		
59	Korea Population, Health and Welfare Association Family Health Clinic	Yun-jeong Kim		
60	Park Jae-young ENT Clinic	Jae-young Park		
61	Bugang Internal Medicine Clinic	Seong-soo Moon		
62	Bae Cheol Clinic	Cheol Bae		
63	Yonsei Open Clinic	Cheol-min Lee		
64	Hyemin Hospital	Chung-hoon Kim		
65	Da Vinci Hospital	Myeong-hoon Kim	P01-202208-01-035	2022-08-25

66	Kim Byeong-cheol ENT Clinic	Byeong-cheol Kim	(Public Institutional Review Board)	
67	Manstone Urology Clinic	Jong-yeon Lee		
68	Han Seung-jin Neurosurgery Clinic	Seung-jin Han		
69	Sintongbangtong Clinic	Jin-seok Oh		
70	Zenit Urology Clinic	Yeong-hwan Jeong		
71	Cho Urology Clinic	Tae-yoon Cho		
72	Joo Young-min Urology Clinic	Young-min Joo		
73	Hwang & Park Psychiatry Clinic	Myeong-won Jeong		
74	Hansarang Family Medicine Clinic	Sang-yoon Park		
75	Kim Gi-young Internal Medicine Clinic	Gi-young Kim		
76	We & Dae Uri Keun Internal Medicine Clinic	Jin-ho Kim		
77	Plus Internal Medicine Clinic	Seung-min Kim		
78	Plus Internal Medicine Clinic	Sae-rom Choi		
79	Kim Nam-dong Internal Medicine Clinic	Nam-dong Kim		
80	Lee Myeong-ho Internal Medicine Clinic	Myeong-ho Lee		
81	Good Morning Clinic	Beom-sik Lee		
82	Kim Cheol-soo ENT Clinic	Cheol-soo Kim		
83	Kwon Tae-gyeong Internal Medicine Clinic	Tae-gyeong Kwon		
84	Dongyang Good Morning Radiology Clinic	Jong-tae Ji		
85	CMI Clinic, Jungang Medical Foundation	Ji-hoon Yang		
86	Bae Ji-ho Neurology Clinic	Ji-ho Bae		
87	Soo Internal Medicine Clinic	Hyeong-won Yang		
88	Dasom Clinic	Myeong-gon Song	P01-202208-01-035	2022-08-25

89	Highman Urology Clinic	Tae-woo Kim	(Public Institutional Review Board)	
90	Seoul Clinic	Yong-beom Kim		
91	Lee Sang-myeong Internal Medicine Clinic	Sang-myeong Lee		
92	Seokdong Internal Medicine & Orthopedic Union Clinic	Jin-cheol Ahn		
93	Yoo Jin-mok Internal Medicine Clinic	Jin-mok Yoo		
94	Kim Du-hwan Internal Medicine Clinic	Si-ho Kim		
95	Seoul Neurology & Internal Medicine Clinic	Young-jae Oh		
96	Choi Jun-ho Internal Medicine Clinic	Jun-ho Choi		
97	Gimhae Centum Hospital	Byeong-hak Cho		
98	Bang Seung-ho Internal Medicine Clinic	Seung-ho Bang		
99	Somang Clinic	Young-soo Kim		
100	Pureun Internal Medicine Clinic	Min-gi Kim		
101	Hue Hospital	So-yeon Kim		
102	Moon Hee-geon Hope Internal Medicine Clinic	Hee-geon Moon		
103	Yang Internal Medicine Clinic	Sang-ho Yang		
104	Pyeonghwa Orthopedic Clinic	Man-seok Ko		
105	New Ulsan Urology Clinic	Min-young Park		
106	Lee Gyeong-hwan ENT Clinic	Gyeong-hwan Lee		
107	Sangji Pureun Clinic	Dong-hyeon Lee		
108	Garam ENT Clinic	Gyeong-seop Noh		
109	Choi Hwan-jun Internal Medicine Clinic	Hwan-jun Choi		
110	The Sarang Clinic	Do-yeon Kim		
111	Ahn Byeong-hyeon ENT Clinic	Byeong-hyeon Ahn	P01-202208-01-035	2022-08-25

112	Health First Clinic	Seong-hyeon Park	(Public Institutional Review Board)	
113	Heo Yong Family Medicine Clinic	Yong Heo		
114	Haengbokhan Clinic	Wan-nam Kang		
115	Gil ENT Clinic	Jong-gil Lee		
116	Yoo Jun-sang Family Medicine Clinic	Jun-sang Yoo		
117	Neulpureun Uri Internal Medicine Clinic	Byeong-gon Kim		
118	Baek Kang-woo Internal Medicine Clinic	Kang-woo Baek		
119	Wisaeng Internal Medicine Clinic	Seung-seok Ko		
120	Bright Internal Medicine Clinic	Jeong-il Kim		
121	Wando ENT Clinic	Young-sik Noh		
122	Sijong Kim Clinic	Chang-nam Kim		
123	Columban Clinic	Jun Choi		
124	Daehan Family Medicine Clinic	Sang-hyo Song		
125	Park Young-bu Internal Medicine Clinic	Young-bu Park		
126	Singil Family Medicine Clinic	Chang-hoon Nam		
127	Yeongtong Top Internal Medicine Clinic	Jong-seol Park		
128	Hyeonje Clinic	Yeong-sik Hyeon		
129	Yonsei Haesol Family Medicine Clinic	Hyo-jeong Kim		
130	Manduk Soksiwon Internal Medicine Clinic	Gyeong-jin Kim		
131	Manduk Soksiwon Internal Medicine Clinic	Gwang-jae Lee		
132	Seongmo Union Clinic	Jae-nam Ma		
133	Doori ENT Clinic	Eun-seok Lim		
134	Park Nam-il Urology Clinic	Nam-il Park	P01-202208-01-035	2022-08-25

135	Mirae Internal Medicine Clinic	Byeong-hyeon In	(Public Institutional Review Board)	
136	Kang Gyeong-tae Internal Medicine Clinic	Gyeong-tae Kang		
137	Park Min-ho Internal Medicine Clinic	Min-ho Park		
138	Fatima Clinic	Jong-oh Lee		
139	Geochang Samsung Internal Medicine Clinic	Min-soo Kim		
140	Kang Internal Medicine & Seogang Yakson Korean Medicine Clinic	Seong-ju Kang		
141	Wibareun Internal Medicine Clinic	Gyeong-ho Yang		
142	Asan Park Internal Medicine Clinic	Moon Park		
143	Jeil Internal Medicine Clinic	Geon-yeong Kim		
144	Shin Internal Medicine Clinic	Yong-min Shin		
145	Doori ENT Clinic	Myeong-ho Jeong		
146	Jeil Internal Medicine Clinic	Heon-jin Park		
147	Dream ENT Clinic	Hyeon-soo Kim		
148	Shin Gyu-chang Prime Union Clinic	Gyu-chang Shin		
149	Yonsei Family Medicine Clinic	Yong-pyo Jeong		
150	Kim Yong-tae Internal Medicine Clinic	Yong-tae Kim		
151	Goryeo Family Medicine Clinic	Seong-tae Bae		
152	Yonsei S Hospital	Tae-hoon Kim	2022-02	2022-09-22
153	Chungnam National University Hospital	Hee-seok Moon	2022-11-054	2022-12-02
154	Korea University Ansan Hospital	Do-hoon Kim	2022AS0296	2022-11-15
155	SMG-SNU Boramae Medical Center	Ji-won Kim	10-2022-126	2023-01-20
156	Kangwon National University Hospital	Bong-ki Lee	2022-12-009	2023-01-02

157	Daedong Hospital	Chang-seop Lim	2023-03	2023-05-24
158	Sejong Chungnam National University Hospital	Ju-seok Kim	2022-12-011	2022-12-23
159	Good Gang-an Hospital, Eunseong Medical Foundation	Dong-hyun Lee	GGAH 2023-02	2023-02-22
160	Korea University Guro Hospital	Seung-han Kim	2023GR0071	2023-02-23
161	Soonchunhyang University Cheonan Hospital	Sang-ho Park	SCHA2023-04-039	2023-04-26
162	Inje University Ilsan Paik Hospital	Jun-hyuk Son	ISPAIK-2023-05-010	2023-06-12
163	Korea University Guro Hospital	Dong-oh Kang	2023GR0367	2023-08-28
164	Korea University Guro Hospital	Beom-jae Lee	2023GR0421	2023-09-21
165	Sarang General Hospital, Chilseok Medical Foundation	Young-hee Lim	2023001	2023-09-15
166	Pusan National University Yangsan Hospital	Cheol-woong Choi	12-2023-003	2023-11-09
